# Non-disclosure of HIV-positive status to a partner and mother-to-child transmission of HIV: Evidence from a case–control study conducted in a rural county in Kenya

**DOI:** 10.4102/sajhivmed.v18i1.691

**Published:** 2017-11-29

**Authors:** Joram Nyandat, Gisela van Rensburg

**Affiliations:** 1 Department of Health Studies, University of South Africa, South Africa

## Abstract

**Background:**

Many factors contribute to an enhanced risk of infant HIV acquisition, two of which may include failure of a mother to disclose her HIV-positive status to her partner and exclusion of male partners in preventing mother-to-child transmission of HIV (PMTCT) interventions. To justify why HIV programmes need to integrate male partner involvement and partner disclosure, we need to establish an association between the two factors and infant HIV acquisition.

**Objective:**

To determine whether failure to disclose an HIV-positive status to a male partner is associated with increased risk of infant HIV acquisition, and whether part of the association is explained by exclusion of male partner in PMTCT programmes.

**Methods:**

Using a case–control study design, we identified a total of 180 mother–baby pairs with HIV-exposed infants. Thirty-six pairs with HIV-positive babies (cases) were compared to 144 pairs with HIV-negative babies (controls) on whether the mothers had disclosed their HIV status to their partner in order to determine whether a disclosure or lack of it contributed to increased risk of mother-to-child transmission of HIV (MTCT). Each case pair was matched to four control pairs from the same facility.

**Results:**

Overall, 16.7% of mothers had not disclosed their HIV status to their partners, the proportion being significantly more among cases (52.8% vs. 7.6%, *p* < 0.001). Non-disclosure was significantly associated with infant HIV acquisition (aOR 9.8 (3.0–26.3); *p* < 0.001) and male partner involvement partially mediated the effect of non-disclosure on infant HIV acquisition (indirect coefficient = 0.17, *p* < 0.005).

**Conclusions:**

Failure of an HIV-positive woman to disclose her status to her male partner and exclusion of male partners in PMTCT programmes are two social factors that may curtail success of interventions towards the goal of eliminating MTCT.

## Introduction

Transmission of HIV from the mother to the child (vertical transmission) can occur across the placenta during pregnancy, during the process of delivery and after birth through breast milk.^1^ Breastfeeding and antiretroviral intake modify the risk of vertical transmission. The risk of vertical transmission among mothers not on antiretroviral treatment (ART) in non-breastfeeding population ranges from 15% to 30%, with the risk being higher among breastfeeding women (20% – 45%).^2^ In high-income countries, because of universal access to interventions aimed at preventing mother-to-child transmission of HIV (PMTCT), the risk of vertical transmission has been reduced to less than 1%.^3^ Globally, PMTCT interventions have resulted in a drastic decline in the incidence of HIV among children. By 2013, 58% fewer children were infected when compared to infection rates in 2002. According to the United Nations Children’s Fund (UNICEF) data sheet,^4^ approximately 150 000 new HIV infections occurred in 2015, which is a significant improvement from the 2009 statistic of 330 000 new infections. However, despite the achievement, the number of new childhood infections is still high considering that PMTCT interventions are universally available. UNICEF^4^ estimates that approximately 6600 children in Kenya were infected with HIV in 2015, representing a mother-to-child transmission of HIV (MTCT) rate of 8%. A study by Nyandiko et al. in 2013 reported MTCT rates of 4% – 8% among a population in western Kenya.^5^ A nationwide survey by Kenya National AIDS and STI Control Programme (NASCOP) reported a higher MTCT rate of 16% among all the infants tested.^6^ The MTCT rates quoted are, however, considered to be conservative because of low uptake of Early Infant Diagnosis, estimated to be around 44%.^4^ The high incidence rate of HIV among children in sub-Saharan Africa has been partly responsible for the reversal of gains witnessed in the past 15 years towards reducing childhood mortality.^7^ More concerted effort is thus required if the goal of eliminating MTCT (EMTCT) is to be realised.

Kenyan PMTCT guidelines (2013) required that any HIV pregnant mother be put on Highly Active Antiretroviral therapy (HAART) during pregnancy irrespective of stage of disease, CD4 count or viral load. On delivery, the infant would receive nevirapine prophylaxis for a period of six weeks. The mother–baby pair was then followed up for a period of 18 months during which the infant would be tested for HIV using DNA polymerase chain reaction (PCR) at six weeks of age, at 9 months and an antibody-based test at 18 months of age. The follow-up of the mother–baby pair was documented in a cohort register called the mother–baby pair register.^8^

While currently, focus has shifted towards addressing psychosocial determinants of vertical transmission, at the beginning of the pandemic when access to antiretroviral drugs was limited, clinical factors were the major risk factors for MTCT. Maternal stage of HIV infection as demonstrated by CD4 count and viral load was majorly responsible for vertical HIV transmission, and undetectable viral load (viral load of < 20 copies/mL) was protective against MTCT.^9^ Antenatal use of ART reduces risk of vertical transmission of HIV by inhibiting viral replication.^10^ The best route of delivery for prevention of MTCT remains controversial. Caesarean delivery before onset of labour has, however, been shown to present a lower risk of MTCT as compared with vaginal delivery.^11^ Torpey et al.^12^, however, reported that the risk of transmission with vaginal delivery is comparable to that of caesarean delivery if both mother and baby are on ART and prophylaxis respectively. However, the risk increases if invasive intrapartum procedures such as episiotomies are used.^13^ Factors associated with the baby that may increase the risk of acquiring HIV include small for gestational age, low birth weight and failure to receive antiretroviral (ARV) prophylaxis.^9,14,15^

Uptake and utilisation of PMTCT interventions present a proven and effective social strategy for EMTCT, with antenatal HIV counselling and testing, disclosure of HIV-positive status to a partner and inclusion of the male partner underpinning the success of PMTCT interventions in reducing risk of vertical transmission.^16^ In keeping with World Health Organization (WHO) recommendation to routinely test and treat all pregnant women with HIV as part of antenatal care, an increasing number of pregnant women are being tested for HIV. Consequently, a greater number of pregnant women are aware of their HIV status.^17^ In Kenya, awareness of HIV status among pregnant women stands at 66%.^18^ However, the increased awareness of HIV status has not translated into more male partners being disclosed to, more so if the woman is HIV-infected.^19,20^ Several advantages of HIV-positive women disclosing their status have been well described. Disclosure has been shown to increase the level of support towards the pregnant woman, including increased involvement in healthcare processes such as PMTCT interventions.^21^ More specifically, disclosure increases uptake and adherence to antiretroviral drugs (for both mother and baby) for the purpose of PMTCT.^21,22^ This is because HIV-positive pregnant women are at risk of stopping their ARVs to avoid accidental disclosure to the partner. Disclosure also encourages uptake of and adherence to appropriate infant feeding option. In an African set-up, there may be increased pressure by the extended family for early initiation of mixed feeding. Moreover, should the mother prefer formula feeding, the pressure exerted by the family would not make it possible to follow through the option. Disclosure releases the pressure of early mixed feeding.^23^ Finally, women who have disclosed their HIV-positive status to their partners are more likely to deliver at a health facility and will in the process receive appropriate PMTCT interventions.^24,25^

Despite the benefits of disclosure to MTCT, disclosure rates are still low. A systematic review of disclosure rates by Medley et al.^26^ reported varying rates between 16.7% and 86%. Low disclosure rates have been reported in some parts of Tanzania (41%),^27^ Kenya (49%)^28^ and Zimbabwe (66%).^29^ However, disclosure rates were high in Nigeria (90%), Zimbabwe (97%) and in Namibia, some parts of Tanzania and Ethiopia (80%).^30,31,32^ Variable rates in disclosure justify focussing on non-disclosure as a key behavioural risk factor for MTCT.^33^

Partner disclosure among women attending antenatal clinics can be promoted by implementing couple testing programmes. Couple testing removes the burden of disclosure on the woman as it encourages mutual disclosure between partners.^34,35^ Other factors positively correlated with disclosure include status of relationship, with married women in a stable relationship being more likely to disclose.^36,37^ Awareness of partners’ HIV status also reduces chances of disclosing one’s own status by 98%.^38^ Additionally, younger women with no children and more educated women are less likely to disclose.^27,38,39^ Finally, a woman on antiretroviral medicines is more likely to have disclosed her status as pre-treatment counselling emphasises disclosure as part of adherence counselling.^40^

Limited studies have been conducted to evaluate how non-disclosure to a male partner affects risk of MTCT. Moreover, results reported have been contradictory.^21,22,28,41^ This study was based on the background of these inconsistent findings and on the scarcity of studies on the association between non-disclosure of HIV-positive status to partners and MTCT. We therefore sought to examine the relationship between non-disclosure of an HIV-positive status to a partner and infant HIV acquisition. Further to finding an association we sought to determine whether the effect of non-disclosure on MTCT is partially related to male partner involvement.

## Materials and methods

### Study design

Using a case–control study design, we identified a total of 180 mother–baby pairs with HIV-exposed infants (HEIs). Thirty-six pairs with HIV-positive babies (cases) were compared to 144 pairs with HIV-negative babies (controls) on whether the mothers had disclosed their HIV status to their partner (exposure) in order to determine whether disclosure or lack of it contributed to increased risk of MTCT. Each case pair was matched to four control pairs from the same facility.

### Study setting

The study was conducted in Siaya County, Kenya. Siaya County, one of the 47 counties in Kenya, is located in western Kenya, to the east of Lake Victoria. The county has six sub-counties, and as of 2016, had an estimated population of 932, 754. Approximately 54% are females (500,889) and has one of the highest HIV prevalence rates in Kenya at 24.8%, and an equally high MTCT rate of 21%.^42^ Similarly, the high childhood mortality rates (partly attributed to HIV-related complications and malnutrition) of 39/1000, 111/1000 and 159/1000 for neonatal mortality rate, infant mortality rate and under five mortality rates respectively are some of the highest in Kenya.

The public health system in Kenya follows a hierarchical pyramidal structure with four levels (also called tiers) of service delivery. Community units form the lowest level of service delivery (tier 1), focussing on the promotion of appropriate healthy behaviours. This level has no physical structures. Above this level are primary care facilities (dispensaries, health centres, clinics and maternity homes) whose focus is on disease prevention and health promotion services. County referral hospitals (tier 3) encompass county and sub-county referral facilities. Tier 3 facilities not only provide comprehensive in-patient diagnostic, medical, surgical and rehabilitative care, but also facilitates referrals to the National Hospitals, which form the highest level of health service delivery in the county (tier 4).^42^

The highest level of care in the county is provided by the County Referral Hospital. Siaya County has three tier 3 health facilities and 154 tier 2 health facilities.^42^ Participants for the study were enrolled from the County Referral Hospital and the five sub-county referral hospitals (Tier 3 facilities). The selection was based on the fact that tier 1 and 2 facilities do not normally conduct deliveries. Sub-county and the county referral hospitals deliver more than 95% of babies in the county, the remaining 5% being handled by health centres and private facilities in the county.^42^

### Participants

The target population for this study was HIV-positive mothers and their infants (referred to in this study as a mother–baby pair). A mother–baby pair being cared for at a health facility in Siaya County became eligible for enrolment into the study. Additionally, the infant in the pair should have been born between January 2013 and June 2014 in any of the five sub-county and one county referral hospitals.

To identify eligible mother–baby pairs, we reviewed the mother–baby pair HIV cohort registers provided by the Ministry of Health to all health facilities providing PMTCT services. Identified HIV-positive mother–baby pairs provided the sampling frame. Incidence density sampling was used in the selection of cases, and Simple random sampling was used to select ‘potential’ controls from the sampling frame. We aimed to select four controls for every case identified. A case was identified as an HIV-positive mother–baby pair with an infant who tested HIV-positive at six weeks of age and since cases were newly diagnosed, they represented incident cases for the study period. A control was an HIV-positive mother–baby pair with an infant who was HIV-negative at six weeks. Controls were individually matched to cases by the health facility. We excluded from the study mother–baby pairs with mothers who were not in a sexual relationship during the pregnancy and postnatal period.

Health facilities within the county serve a defined geographic region with a specific catchment population. The population differs on economic status, facility–population ratio, customs and traditions, and health-seeking behaviours. Moreover, health facilities vary in terms of standards of care, drug stock outs (in reference to ARVs) and practices. All these factors are likely to affect disclosure as well as MTCT. Therefore to control for these variables *a priori*, we matched one case to four controls in a given facility.

### Variables

Included in this analysis were mother–baby pairs composed of HEIs and their mothers. The definition of HEI was adopted from the Ministry of Health, Kenya, which defines an HEI as a child (who is less than 18 months old) born to an HIV-positive mother. In this study, any mother with an HIV-positive status, as recorded in the HEI facility, book was taken to be HIV-positive. Our exposure variable was non-disclosure of HIV status to a partner, which in this study was defined as failure by an HIV-positive mother to disclose her positive status to her partner prior to learning the infant’s HIV status. Mothers were asked whether they had disclosed to their male partners prior to knowing the 6-week test outcome done at the health facility. The test is usually undertaken during the first immunisation schedule, and this was used as an ‘aide de memoire’. A male partner was defined as presence of a male figure in the life of the baby with whom the mother was having a sexual relationship prior to knowing the HIV status of the baby. Male partner involvement was defined as male partner accompanying the woman to the hospital at any time during the pregnancy, delivery and 6-week immunisation visit.

**FIGURE 1:**
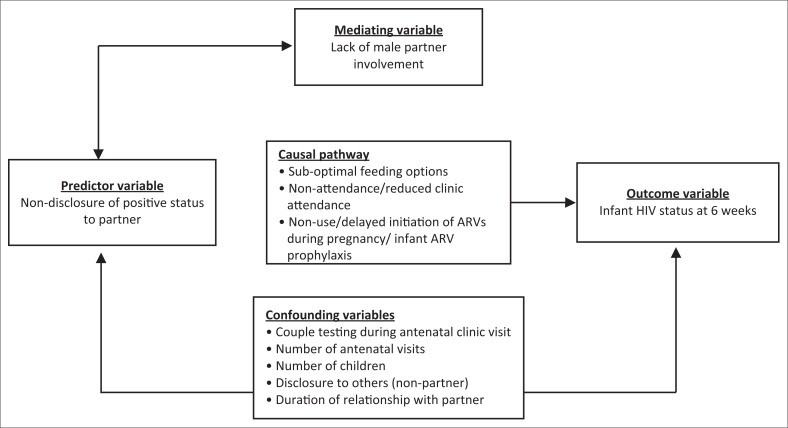
Conceptual framework for relationship among variables affecting non-disclosure and mother-to-child transmission of HIV.

Non-disclosure of HIV-positive status to male partner was hypothesised to contribute to the risk of infant HIV acquisition. We further hypothesised that male partner involvement is partially responsible for the observed effect of non-disclosure on infant HIV acquisition by determining uptake of PMTCT interventions such as hospital visits for PMTCT, uptake of ART and infant prophylaxis and adherence to safe feeding option for the infant. These variables were considered to be in the causal pathway as illustrated in Figure 1. In the statistical analyses, factors considered potential confounders and not matched for were couple testing, disclosure to others, length of relationship with partner and number of antenatal visits.

### Data source and measurement

The county’s HIV-positive mother–baby pair register was used to identify HIV-positive mother–baby pairs receiving care at the health facility. The 6-week HIV PCR result, also documented in the mother–baby pair register, was used to classify the mother–baby pairs into cases (mother–baby pair with an infant whose 6-week HIV PCR test was positive) or controls (mother–baby pair with an infant whose 6-week HIV PCR test was negative). We then retrieved the medical records of the potential respondents (defined as mothers in the mother–baby pair), which allowed us to contact potential respondents, describe the purpose of the study and request them to consider being part of the study. Mothers who showed willingness to be included in the study were enrolled and presented with questionnaire during their next routine clinical visit. During the visit, the aim of the study was explained to the mothers again, after which a signed written consent was obtained before the mothers were given the questionnaire to complete. The researcher was at hand to clarify and guide the respondents when required. The questionnaire extracted information related to mother’s personal details, status of disclosure to the partner or others, couple testing, awareness of HIV status of the partner and the length of relationship. Male partner involvement was considered by collecting information on accompaniment by the partner to the clinic during pregnancy and after delivery, and couple testing.

### Bias

Threats to validity were well anticipated and addressed. Selection bias was minimised by using results from PCR test which has a high sensitivity (100% at one month of age) and specificity (99.8% at birth and 100% at one month of age).^43^ Recall bias because of likelihood of cases remembering exposures to risk factors more than controls was minimised by limiting the time period of exposure to less than two years. The questionnaire was self-administered, minimising risk of bias from data collectors.^44^

### Sample size

The study required a sample size of 34 case–control sets. Using a difference in proportions formula for matched case–control studies,^45^ we assumed a mean proportion of non-disclosure of 40% and 15% in case and control respectively, an odds ratio of two for a case–control matched ratio of 1:4, a power of 80% and significance level of 5%.

### Statistical methods

Normality was assessed visually using box-plots and statistically using Shapiro–Wilk’s test (*p* < 0.05).^46^ To determine whether there were differences between case and control groups with regard to study variables, we employed student’s *t*-test for continuous data and chi-square test for categorical data as required. The odds ratio, 95% confidence intervals and significance level of the matched-pair data were calculated using Mantel–Haenszel statistical test. The significance level was set at *p* < 0.05. Variables which were significant in bivariate analysis and not in the causal pathway were controlled for in the multivariable analysis using conditional logistic regression.

**FIGURE 2:**
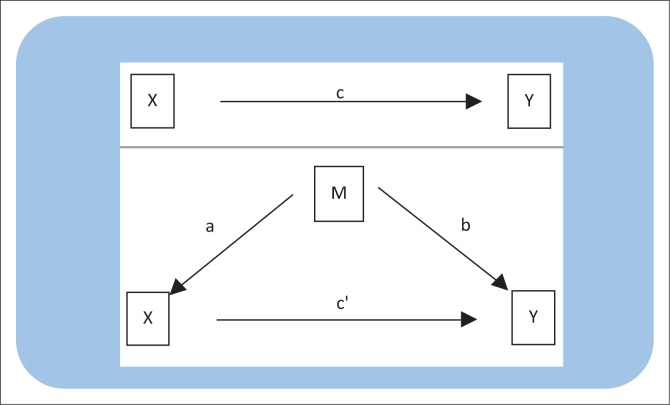
Framework for mediation analysis^47^.

To determine mediation, we performed four regression analyses as illustrated in Figure 2. The first analysis assessed whether non-disclosure (independent variable) is significantly associated with MTCT (dependent variable). This analysis is illustrated in the diagram as path c. The second analysis assessed whether non-disclosure was significantly associated with male partner involvement (mediator variable) illustrated in the diagram as path a. The third analysis assessed whether male partner involvement is associated with MTCT (dependent variable) illustrated as path b. Finally, to determine partial versus full mediator effect, we controlled for the effect of male partner involvement on MTCT illustrated as path c′.^47^

Statistical Package for Social Sciences (SPSS) version 22.0 for Windows was used for analysis. STATA v12 and MPLUS (Version 6.11) were used for matched analysis, and significance of the mediation tested using the Sobel test.^48^

## Ethical consideration

Ethical and administrative approval of the study methodology was granted by the Research and Ethics Committee, Department of Health Studies, UNISA, on 29 October 2013 and local ethical approval obtained from Moi University, College of Health Sciences Research and Ethics Committee on 03 March 2014. Permission to conduct the study in Siaya County was sought and granted by the County Director of Health. All facilities received a copy of the research proposal and copies of the ethical clearance certificates together with the request for permission to conduct the study at the institution. Additional verbal permission was obtained from the in-charges of the various health facilities. Written, informed consent was obtained from each participant prior to the interviews.

## Results

### Response rate

**FIGURE 3:**
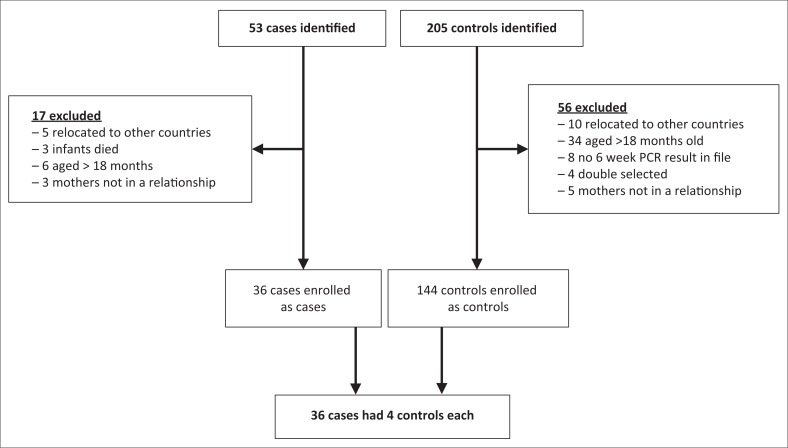
Summary of inclusion and exclusion of participants.

The response rate of 72% was deemed adequate as it represented the minimum sample size required to achieve an 80% power.^49,50^ Fifty three cases and 205 controls were identified in the study, of which 36 cases and 144 controls were enrolled in the study (Figure 3). The main reasons for non-participation were relocation (cases 32%, controls < 18%), infants aged more than six months (cases 35%, controls 61%) and mother not in any relationship (cases 18%, controls 9%). Other reasons included death of infants (17% cases) and lack of PCR result in register (control 14%). There was no missing information as we ensured completeness of the filled questionnaire. We compared mother–baby pairs not enrolled to the study to those enrolled in the study with regard to three variables (age of mother, birth weight of infant, HIV disclosure) to determine whether the two groups differed on the selected variables. (Table 1) The information for non-enrolled pairs was obtained from the mother–baby pair cohort register. A two-tailed, two-sample *t*-test was used to compare mother’s mean age and child’s birth weight, and chi-square test was used to compare disclosure status. As shown in Table 1, the differences were not statistically significant at the 0.05 level of significance. Therefore, we were confident of minimal error of effect measures obtained from the study.^51^

**TABLE 1:** Differences in characteristics of respondents versus non-respondents.

Variable	Respondents (*n* = 180)				Non-respondents (*n* = 78)				*p*
	*N*	%	Mean	SD	*N*	%	Mean	SD	
Mother’s age (years)	-	-	27.4	5.4	-	-	26.9	4.2	0.327
Child’s birth weight (kg)	-	-	3.2	0.6	-	-	2.9	1.3	0.289
Disclosure status† (yes)	150	83.0	-	-	55	78.0	-	-	0.642

† , Disclosure status for non-respondents was obtained from medical records at the facility.

### Descriptive statistics

Overall, 43% (*n* = 78) of the infants in the mother–baby pairs were female and the median (IQR) age was 10 (7) months. The mean (SD) birth weight was 3.2 (0.6) kg and the mean (SD) maternal age was 27.4 (5.4). Sixty-six per cent (*n* = 119) of mothers were in monogamous relationship compared to 24% (*n* = 29) in a polygamous relationship. Most mothers (78.3%) had primary school education, with 21.6% (*n* = 39) having obtained post-primary school education.

### Non-disclosure and mother-to-child transmission of HIV

Approximately 17% (*n* = 30) of mothers in the mother–baby pairs had not disclosed their HIV-positive status to their partners. A significantly higher proportion of cases had not disclosed their HIV status (52.8% vs. 7.6%; OR = 11.8 [4.5–30.8]). Four variables were significantly found to be associated with MTCT. Infant prophylaxis and absolute breastfeeding were significantly associated with MTCT (OR = 0.12 [0–0.9]) and 0.19 (0.07–0.48), respectively. Involvement of the male partner and awareness of HIV status of the partner were also associated with infant HIV acquisition (OR = 0.30 [0.10–0.50] and OR = 0.12 [0.1–0.90]) (Table 2). Awareness of male partner status was the only variable significantly associated with infant HIV acquisition and not in the causal pathway. During multivariable analysis, even after controlling for awareness of male partner HIV status, non-disclosure of HIV-positive status to a partner maintained its significance as a risk factor for MTCT aOR 9.8 (95% CI 3.0–26.3) (Table 3).

**TABLE 2:** Comparison of variables between cases and controls.

Variable	Category	Total		Case		Control		OR	95% CI	*p*
		*n*	%	*n*	%	*n*	%			
Marital status	Unmarried	18	10	5	13.2	13	9.2	-	-	0.45
	Married, monogamy	119	66	28	73.7	91	64.1			
	Married, polygamy	29	16	4	10.5	25	17.6			
	Divorced or widowed	14	7.8	1	2.6	13	9.1			
Educational level	Basic	141	78.3	31	81.6	110	77.5	-	-	0.64
	Post-primary	39	21.6	7	18.4	32	22.5			
Age of child	< 6 months	39	21.7	11	28.9	28	19.7	-	-	0.22
	>6 months	141	78.3	27	71.1	114	80.3			
Mother’s age	<25	59	32.8	16	42.1%	43	30.3	-	-	0.11
	>25	121	67.2	22	57.9	99	69.7			
Infant prophylaxis	Yes	174	96.7	34	89.5	140	98.6	0.12	0–0.90	0.005*
	No	6	3.3	4	10.5	2	1.4			
Place of birth	Health facility	150	83.3	30	78.9	120	84.5	0.60	0.30–1.60	0.35
	Home	30	16.7	8	21.2	22	15.5			
Type of feeding	Breastfeeding	158	87.8	26	68.4	132	93.0	0.19	0.07–0.48	<0.001*
	Mixed feeding	22	12.2	12	31.6	10	7.0			
Mode of delivery	No procedure	172	95.6	38	100	134	94.4	-	-	0.35
	Procedure	8	4.4	0	0	8	5.6			
Antenatal clinic attendance	Yes	179	99.4	38	100	141	99.3	-	-	0.80
	No	1	0.6	0	0	1	0.7			
Partner involvement	Yes	33	18.3	4	10.5	29	20.4	0.12	0.10–0.90	<0.001*
	No	147	81.7	34	89.5	113	79.6			
Maternal prophylaxis	Yes	165	91.7	32	84.2	133	93.7	0.39	0.13–1.19	0.09
	No	15	8.3	6	15.8	9	6.3			
Duration aware of status	<1 year	55	30.6	9	23.7	46	32.4	0.7	0.3–1.7	0.425
	>1 year	125	69.4	29	76.3	96	67.6			
Duration of relationship with partner	<1 year	156	86.6	32	83.8	124	87.3	0.8	0.2–2.3	0.713
	>1 year	24	13.4	6	16.2	18	12.7			
Time of disclosure	Before pregnancy	143	93.5	18	85.7	125	94.7	0.2	0.1–1.4	0.112
	After pregnancy	10	6.5	3	14.3	7	5.3			
Time to disclosure	<3 months	116	75.8	17	85	99	77.4	1	0.2–4.0	1.000
	>3 months	37	24.2	3	15	34	25.6			
Awareness of partner status	Yes	109	60.6	14	36.8	95	66.9	0.3	0.1–0.5	<0.001*
	No	71	39.4	24	63.2	47	33.1			
Tested as a couple	Yes	43	23.9	6	15.8	37	26.1	0.56	0.23–1.36	0.201
	No	137	76.1	32	84.2	105	73.9			
Disclosed to other people	Yes	135	75.0	27	71.1	108	76.1	0.8	0.3–1.7	0.528
	No	45	25	11	28.9	34	23.9			
Encouraged to disclose by health worker	Yes	167	92.8	36	94.7	131	92.3	1.7	0.3–8.0	0.494
	No	13	7.2	2	5.3	11	7.7			
Follow-up on disclosure	Yes	160	88.9	33	86.8	127	89.4	0.9	0.2–2.7	0.809
	No	20	11.1	5	13.2	15	10.6			

OR, odds ratio; CI, confidence interval.

* , *p* = 0.05.

**TABLE 3:** Bivariable and multivariable analysis of non-disclosure of HIV-positive status among cases and controls.

Cases	Control 1			Control 2			Control 3			Control 4			UOR	CI	*p*	AOR	CI†	*p*
	+	-	T	+	-	T	+	-	T	+	-	T						
Exposed	1	18	19	0	19	19	1	18	19	1	16	17	11.8	4.5–30.8	< 0.001	9.8	3.0–26.3	< 0.001
Unexposed	4	13	17	4	16	20	2	15	17	0	17	17						
**Total**	5	31	36	4	35	39	3	33	36	1	33	34	-	-	-	-	-	-

UOR, unadjusted odds ratio; AOR, adjusted odds ratio; CI, confidence interval; T, total +, exposed; -, unexposed.

† , Adjusted for awareness of male partner HIV status.

**FIGURE 4:**
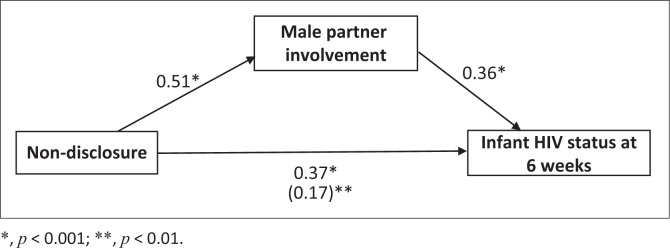
Regression coefficients for relationship between non-disclosure and HIV status at 6 weeks as mediated by male partner involvement.

For mediation analysis, path c regression coefficient illustrated that non-disclosure was significantly associated to MTCT (0.37, *p* < 0.001), satisfying the first condition for mediation (Figure 4). Similarly, path a and path b regression coefficients were significantly related (0.51, *p* < 0.001 and 0.36, *p* < 0.001) satisfying the second and third conditions for mediation respectively. The indirect effect (path c′) suggested partial mediation of male partner involvement on the association between non-disclosure and MTCT as the coefficient was substantially reduced (0.17, *p* = 0.01) compared to the direct effect coefficient of 0.37.

## Discussion

Two significant findings have emerged from this research effort. First is that infants born to HIV-positive mothers are more likely to become HIV-positive if they have a mother who has failed to disclose her HIV-positive status, and second is that the association between non-disclosure and infant HIV acquisition is partly because of the effect of male partner involvement.

Approximately 83.3% of HIV-positive mothers enrolled in the study reported having disclosed to their partner. This proportion is comparable to most reports from sub-Saharan Africa, with disclosure proportions between 16.7% and 86%.^26^ Bachanas et al.^19^ reported 80% of the 3538 HIV-positive patients in Kenya, Tanzania and Botswana had disclosed their status to their partners. Similarly, 70% of 20 HIV-positive pregnant women in Kenya had disclosed their status.^52^ The rate of HIV status disclosure in this study is considered to be conservative. Similar to other similar studies, status of disclosure is based on self-report which raises the possibility of social desirability bias. To minimise reports of disclosure while it did not happen, the researcher did not emphasise any question during data collection to ensure that respondents did not feel some of the questions were more important. We also corroborated the responses obtained from the respondents with the disclosure status documented in the patient’s file. Where there was discordance or when no response was recorded, the mother was contacted to clarify the true position.

One finding unique to this study relates to the significantly higher proportion of HIV-positive infants having mothers who have not disclosed their positive status to their partners. Approximately 52% had not disclosed their status, compared to only 7.6% among mothers with HIV-negative infants (OR = 11.8 [95% CI 4.5–30.9]). The significance of the association was not altered even after controlling for awareness of partner status.

A mother’s decision against disclosing her HIV-positive status to her partner may be motivated by several factors. Central is the ability to overcome difficulty associated with fear of abandonment, accusation of infidelity, discrimination, violence and loss of spousal financial support.^35,53^ The fears experienced by the mothers are justified. Farquhar et al.^35^ reported that women who work away from home or who are more sexually experienced fear disclosure because of the belief that they will be blamed for infidelity. Women who had previously experienced violence were less likely to disclose their HIV status to their partners.^26^ Disclosure is also influenced by the nature of the couple’s relationship, such that disclosure may only be a pointer to the state of the relationship. Married women and partners who have good communication, for example, couples who discussed HIV testing prior to the test, are more likely to disclose their diagnosis to their partner. Length of relationship, level of trust and honesty, and the level of communication within the relationship are just some of the other relationship factors that may influence disclosure.^54^

Recognising multiplicity of factors that inform whether an individual will disclose or not, multiple strategies that address these factors and that promote safe disclosure to a partner ought to be put in place. In sub-Saharan Africa PMTCT guidelines have placed undue burden of disclosure on the tested individual, an approach that has not resulted in satisfactory disclosure rates in many settings. An approach of redistributing the burden of disclosure from the women would theoretically enhance partner disclosure. Facilitated disclosure has a counsellor present during the testing process and they help with disclosure process in the post-testing period. The approach is favoured by HIV-infected couples as it facilitates disclosure, and, not entirely preventing adverse events from occurring, it does reduce adverse events.^52^ Couple testing is a widely used strategy that in addition to removing the burden of disclosure from the woman, it also provides a safe disclosure environment. Couple testing also enhances male partner support for uptake and adherence to PMTCT interventions. Another effective method that is safe and acceptable is couple testing within the comfort of the home. The counsellor is available to support the couple with mutual disclosure.^52^ However, home-based testing may cause stigma consequent to erroneous disclosure to other family members. Finally, it is important to appreciate that gender and societal norms that bar women from having a voice may prevent women from disclosing their HIV-positive status. To address such factors, women’s empowerment programmes and community-based programmes that address stigma associated with HIV can change norms related to gender and in so doing can facilitate positive status disclosure to partners. Support groups for infected women have also been shown to help women in their efforts towards disclosure.

The second important finding also not reported in any of the earlier studies is that involvement of the male partner is partly responsible for the association between non-disclosures of HIV status on MTCT. From previous studies, male involvement has been shown to determine uptake and compliance with PMTCT interventions such as antenatal clinic attendance, adherence to maternal and infant prophylaxis, uptake of appropriate feeding option and reduced number of missed appointments.^21,22,28,34,55,56,57,58,59^ Our study reports that only 18.3% of mothers had male partner involvement. The finding of 18.3% is similar to low rates found in previous studies conducted in Kenya and Tanzania (12.5% and 16% respectively).^60^ Barriers to male partner involvement have been classically divided into health system factors, socio-economic and cultural factors. Health system factors include long hospital waiting times, unfriendly staff and female-dominated services at the hospitals. Culturally antenatal matters have been left to the women and men who get involved have been viewed as being weak. Socio-economic difficulties have discouraged men from getting involved because of time spent at the hospital could be used to work, and the double transport cost implications.^61,62^ The low rate of male partner involvement in this study implies that HIV-positive mothers may lack the support and encouragement of their male partners. Strengthening male partner involvement has the dual effect of enhanced adherence to PMTCT interventions as well as presenting a safe avenue for disclosure of HIV status by removing the burden of disclosure from the woman.^22^

Several African countries have implemented programmes that work with men on various HIV-related issues. These programmes have demonstrated the importance of engaging men in an effort to boost women’s uptake of PMTCT interventions.^55^ Men have been shown to support their involvement in PMTCT and antenatal clinic services.^63^ Strategies to enhance male partner involvement should empower women to have equal responsibility as well as fully participate in decision-making with regard to the health of the family. Health facilities can also support this role by initiating male-friendly services as well as offering services that are exclusively for men. Examples of innovative strategies to enhance male involvement include invitation of male partners to attend either prevention of mother-to-child transmission (PMTCT) or antenatal care (ANC) services using letters addressed to the male partner.^60^ Male involvement may also be enhanced by use of behaviour change as well as communication through the media. The aim of such approaches would be to try and change gender stereotypes as well as educate on involvement in PMTCT. Opinion leaders, such as village elders and pastors, have also been successfully used to deliver educational sessions on importance of male involvement and participation in PMTCT programmes.

### Limitations

The findings from this research effort should be interpreted with the following limitations being considered. Firstly, the generalisability of the findings may be limited to settings with similar socio-demographic, cultural and economic characteristics. Another limitation that may have introduced social desirability bias is the nature of self-reporting for data collection. Self-reports predispose to bias as participants may attempt to present themselves in a perfect manner by lying about questions being asked. This study minimised that possibility by corroborating the reported findings as much as possible with patients’ records, and assuring the respondents of data anonymity, privacy and confidentiality. Another limitation in the study owing to it being a retrospective study is recall bias. It is possible that mothers who had HIV-positive infants were better able to recall when they actually disclosed their HIV status as cases may have a better recall on past exposures than controls. Despite putting in measures to minimise risk of selection bias, facility-based studies are inherently flawed and make it possible that controls selected to represent the population may not be truly representative. In our case, it is possible that pregnant mothers who did not seek care at health facilities may differ from those who were being cared for at health facilities. Finally, mothers who were excluded from the study may have had positive health-seeking behaviours compared to those who were included, possibly introducing bias in the study.

## Conclusion

Evidence from this study suggests an important relationship between failing to disclose HIV-positive status, non-inclusion of male partners’ involvement and infant HIV acquisition. Given the important role played by socio-economic factors in PMTCT, stakeholders need to re-examine policies on disclosure and male partner involvement with a view to addressing the two behavioural contributors to MTCT. Evidence presented should invigorate stakeholders towards identifying and integrating effective methods that address safe disclosure. Additionally, the importance of male partner involvement as a strategy to increase HIV awareness as well as enhance disclosure should be adopted by all PMTCT care providers.

Finally, this study identifies and proposes areas for future research. Despite this study highlighting the associations between MTCT and behavioural variables, more studies need to address other behavioural factors not considered in this study. While we were able to establish that male partner involvement was partially responsible for the association observed between non-disclosure of male partner involvement to a partner and infant HIV acquisition, it is important to point out the simplistic nature of the model tested. More comprehensive mediation analysis on indirect association ought to be performed to fully decipher the complex relationship between MTCT and non-disclosure. A follow-up duplication study in an area with different socio-economic composition and cultural practices, as well as in a metropolitan area, to determine whether the association reported in this current study would hold should be performed.
